# Bibliography

**Published:** 1903-10

**Authors:** 


### Bibliography.

Syphilis in Dentistry. By L. Blake Baldwin M.D., Chicago, Ill., Professor of Dermatology and Venereal Diseases, Post-Graduate Medical School, etc., and Ezra Read Larned, M.D., Chicago, Ill. E. H. Colgrove, Chicago, 1903.

The authors of this book of one hundred and twenty pages, illustrated, dedicate it "To our brothers in arms, the members of the dental profession," and that profession would do well to procure this book and consider seriously its contents. Whether dentists need its lessons more than other members of the healing art is questionable, but that constant familiarity with danger breeds contempt is perhaps more noticeable with members of this calling than with others. It is certainly not true that the present graduate is ignorant of the danger to his patient and himself of infection from this disease, for all are made familiar with it.

It is well, therefore, to keep the danger of infection constantly in view, and the best method of doing this is through text-books of convenient size, and the one under consideration is an excellent example of good text-book making,—clear, concise, and giving all the principal facts connected with the disease in question.

While syphilis, in its many forms, is made more or less familiar to every dental student in clinical cases, it is still a recognized fact that diagnosis is frequently difficult, hence they may inadvertently be led into danger both to themselves and to their patients. The authors of this book give special attention to this possibility, and clearly explain the various forms in which the disease presents.

While the authors keep within the limits of their own specialty, they are upon stable ground, but when they pass over into dentistry, as they do in discussing "interstitial gingivitis," otherwise pyorrhœa alveolaris, they seriously mar the value of their production. The general trend of Part X. is to show "a greater or less interdependence between syphilis and pyorrhœa alveolaris," and yet, in the opinion of the reviewer, no greater mistake could be made than to draw general conclusions from a few isolated cases. That syphilis may produce pericemental inflammations is beyond dispute, but so may other pathological conditions, but pyorrhœa alveolaris, in its general manifestations, is a local irritation. The following quotation condemns the chapter: "Interstitial gingivitis, or pyorrhœa alveolaris, . . . remains to this day a *bête noire* to dentists. This is mainly due to the fact that they cannot cure it, and the best methods at their command to-day lie in prosthetic dentistry." One naturally, after such a sweeping assertion, asks, With whom have the authors mingled in the dental profession? Pyorrhœa alveolaris is very readily cured when occurring between the ages of twenty to fifty, not so readily at later periods.

Aside from this weak chapter, the book can be cordially recommended as a text-book upon this special subject and one worthy of earnest study by practitioners of every grade.

THE FILLING OF TEETH WITH PORCELAIN (Jenkins's System). A Text-Book for Dentists and Students, with one hundred and sixteen Illustrations. By Walter Wolfgang Bruck, D.D.S., Instructor in the Dental Institute of the Royal University of Breslau. Translated from the German by Charles W. Jenkins, D.D.S., Zurich. Consolidated Dental Manufacturing Company, New York.

Dr. Jenkins's method of preparing inlays has been so fully explained by himself and others that it would seem that all in the dental profession must be familiar with the technique of the operation.

The dispute that has been vigorously maintained between the high-fusing and low-fusing advocates seems to be gradually settling down into the conviction that both have a place, and he is the wisest who takes a somewhat eclectic position and selects that best adapted to the case in hand.

The articles published heretofore have been more or less of a fugitive character, or not entering into sufficient detail to be of positive value to the uninstructed. This book, therefore, fills a want as far as the Jenkins system is concerned. The excellent illustrations explain the text very satisfactorily, and it would seem not a difficult matter for any one to follow the methods without having had the benefit of previous personal instruction.

Much credit is due the translator for his portion of the work; it is exceedingly well done.

